# Primary care providers' perspective on prescribing opioids to older adults with chronic non-cancer pain: A qualitative study

**DOI:** 10.1186/1471-2318-11-35

**Published:** 2011-07-14

**Authors:** Aerin Spitz, Alison A Moore, Maria Papaleontiou, Evelyn Granieri, Barbara J Turner, M Carrington Reid

**Affiliations:** 1 Department of Internal Medicine, Virginia Mason Medical Center, Seattle, WA, USA; 2 Division of Geriatrics, David Geffen School of Medicine, University of California, Los Angeles, CA, USA; 3 Division of Endocrinology, University of Michigan School of Medicine, Ann Arbor, MI, USA; 4 Division of Geriatric Medicine and Aging, Columbia University, New York, NY, USA; 5 Department of Internal Medicine, University of Pennsylvania, Philadelphia, PA, USA; 6 Division of Geriatrics and Gerontology, Weill Cornell Medical College, New York, NY, USA

## Abstract

**Background:**

The use of opioid medications as treatment for chronic non-cancer pain remains controversial. Little information is currently available regarding healthcare providers' attitudes and beliefs about this practice among older adults. This study aimed to describe primary care providers' experiences and attitudes towards, as well as perceived barriers and facilitators to prescribing opioids as a treatment for chronic pain among older adults.

**Methods:**

Six focus groups were conducted with a total of 23 physicians and three nurse practitioners from two academically affiliated primary care practices and three community health centers located in New York City. Focus groups were audiotape recorded and transcribed. The data were analyzed using directed content analysis; NVivo software was used to assist in the quantification of identified themes.

**Results:**

Most participants (96%) employed opioids as therapy for some of their older patients with chronic pain, although not as first-line therapy. Providers cited multiple barriers, including fear of causing harm, the subjectivity of pain, lack of education, problems converting between opioids, and stigma. New barriers included patient/family member reluctance to try an opioid and concerns about opioid abuse by family members/caregivers. Studies confirming treatment benefit, validated tools for assessing risk and/or dosing for comorbidities, improved conversion methods, patient education, and peer support could facilitate opioid prescribing. Participants voiced greater comfort using opioids in the setting of delivering palliative or hospice care versus care of patients with chronic pain, and expressed substantial frustration managing chronic pain.

**Conclusions:**

Providers perceive multiple barriers to prescribing opioids to older adults with chronic pain, and use these medications cautiously. Establishing the long-term safety and efficacy of these medications, generating improved prescribing methods, and implementing provider and patient educational interventions could help to improve the management of chronic pain in later life.

## Background

The use of opioid medications to treat chronic non-cancer pain (hereafter referred to as chronic pain) in older patients remains controversial [1-3]. Two recently published guidelines for managing chronic pain [1,2] recommend that clinicians consider opioid therapy for older patients who continue to report moderate-to-severe pain or experience pain-related functional impairment despite non-opioid therapy. However, a recently published comparative safety study with 12,840 participants (mean age = 80 years) suggested that opioids (vs. nonselective and selective nonsteroidal anti-inflammatory medications) confer substantial risks when used to treat older adults with non-cancer pain disorders [3].

Little information is available regarding healthcare providers' attitudes and practices regarding the use of opioids as a treatment for chronic pain in older adults. Provider concerns have been identified in prior studies that did not focus on older adults [4-10]. Concerns identified in these studies included the potential for patient addiction, tolerance and physical dependence [4-10]; abuse, misuse or diversion of opioid medications [4,5,9-12]; inadequate provider training [5,9,12-14]; patient harm from adverse effects [4,6,7,12]; and regulatory sanctions [5-7,9,10]. Age-related changes in cognition, pharmacodynamics and pharmacokinetics, as well as higher rates of comorbidity, polypharmacy, and frailty [15,16] complicate the management of pain in later life, and likely increase providers' concerns about using this class of medications in older patients. Lin et al. [5] surveyed practicing geriatricians and internists to assess for possible provider differences in attitudes regarding the use of opioids as a treatment for patients with chronic pain. Geriatricians voiced far less concern for addiction and more concern for the possibility that patients' pain was being under-treated relative to internists [5]. Provider concerns about causing harm from pharmacologic interventions such as opioid use may contribute to the under-treatment of pain, which is a recognized problem and is associated with advancing age [17]. The Declaration of Montreal, a document developed at the 1st International Pain Summit in 2010, states that "access to pain management by adequately trained health care professionals is a fundamental human right" [18]. Health care providers therefore have an ethical responsibility to relieve pain-related suffering by providing informed and unbiased access to pain medications.

Identifying primary care providers' barriers to the use of opioids as a treatment for chronic pain among older adults could identify possible targets for intervention, and ultimately improve pain management in this expanding patient population. Accordingly, we sought to elucidate primary care providers' perceived barriers and facilitators to prescribing opioids as a treatment for chronic pain among older adults. We also sought to characterize providers' experiences and attitudes towards using this class of medications in older patients.

## Methods

### Design

This was a qualitative, cross-sectional study that employed focus groups to generate discussions among health care providers.

### Study Sites

Focus groups were conducted at the Columbia Presbyterian Allen Pavilion Division of Geriatrics and the NewYork-Presbyterian (NYP) Wright Center on Aging. These sites serve predominantly older Latino and non-Hispanic white patients, respectively. Both practices provide care to patient populations that are older (mean age in the mid 80s) and predominantly female and were selected because they include providers with substantial experience caring for older adults. Many patients seen at the practice have significant functional and/or cognitive impairments. However, most patients live independently in the community, while about 10% reside in assisted-living facilities. Neither practice provides outpatient care to residents of long-term care facilities. Physicians provide longitudinal patient care, while the nurse practitioners see "walk-ins," but do not provide longitudinal care. To increase the diversity of study sites, one focus group was also conducted with providers from three community health centers within the NYP Ambulatory Care Network that serve predominantly Latino patients. In these three practices, virtually all patients live independently in the community. Because the health centers serve patients across a broad range of ages, fewer patients have functional or cognitive deficits relative to the geriatric care practices described above. The study was approved by the Weill Cornell Institutional Review Board.

### Study Participants

All physicians and nurse practitioners (NP) providing care in the above practices were eligible to participate, i.e., no exclusion criteria were employed. Recruitment at the Allen Pavilion and the Wright Center for volunteers occurred during routine staff meetings. Participants were recruited based upon their interest and availability to attend one of the focus group sessions. Each participant selected a time that was convenient from the groups offered. Medical directors at the three community health centers were contacted to recruit providers at these practices. Of 38 eligible providers at the five practices, 26 (68%) participated in a focus group. Participants attended a focus group that was convenient for them. There was no attempt to ensure homogeneity of participants (e.g., in terms of gender, age, years of clinical experience) across the various groups. Of the 12 providers who did not participate, all cited scheduling conflicts as a reason for not participating. Participants did not differ from nonparticipants with respect to age, gender, race/ethnicity status, or years of training.

### Focus Group Methodology

One of two investigators (MCR or AS) introduced the study, explained the focus group objectives and process and then moderated a semi-structured discussion using open-ended questions and follow-up probes that were created based upon a review of the literature and pilot tested prior to use in the current study. Providers were asked to share their experiences using opioids in older adults with chronic pain, characterize their use of these medications, describe perceived barriers and facilitators to prescribing opioids prescribing to their older patients, voice any legal/regulatory or abuse/misuse concerns, and finally describe their comfort level(s) prescribing opioids for older patients with chronic pain and those receiving palliative or hospice care. Demographic and practice-relevant data were collected at the end of each focus group via a self-administered questionnaire.

### Analysis

All focus groups were audiotape recorded, transcribed, and analyzed via directed content analysis [19]. The qualitative data were analyzed after each focus group. The transcripts were read and then preliminary themes were generated taking into consideration themes identified in previous studies (e.g., barriers to opioid prescribing), as well as newly identified themes. The basic unit of analysis consisted of discrete sections of text that were felt to convey an idea or construct by the investigators. For example the phrase, "A lot of my patients don't want to take opioids, which is a big impediment to prescribing them," was coded as "patient reluctant to take opioid." Two investigators (AS, MCR) reviewed the focus group data independently and then met to discuss observations and reconcile divergent interpretations until agreeing upon a single common set of themes. The transcripts were again reviewed and the data from the common set of themes were entered into QSR NVivo 8 [20] to facilitate theme sorting and counting. Focus groups were conducted until the investigators agreed that the discussions produced no new themes, i.e., thematic saturation was reached.

## Results

### Focus Groups and Participants

Six provider focus groups were conducted, ranging in size from three to eight participants and lasting from 35 to 60 minutes. Table 1 shows that providers (23 physicians and 3 nurse practitioners) had a mean age of 40 years, were mostly female (77%), and reported an average of 12 years in practice.

**Table 1:** Study Participants' Characteristics

Characteristics	Total (N = 26)
Mean age, years (range)	40 (28-60)
Female, n (%)	20 (77)
Non-Hispanic white, n (%)	14 (54)
Provider type, n (%)*	
Physician	23 (88)
Nurse practitioner	3 (12)
Physicians (n = 23) with geriatric fellowship training, n (%)	21 (91)
Mean number of years in practice, n (range)	12 (4-39)
More than 50% of time in direct patient care, n (%)	22 (87)
More than 75% of patients above age 65, n (%)^†^	20 (77)
Residence status of practice patients, (%)	
Independent	(90)
Assisted-living	(10)
Percentage of older patients with chronic pain, n (%)^†^	
<25%	10 (38)
26-50%	9 (35)
51-75%	5 (19)
>75%	1 (4)
Percentage of older patients with chronic pain on an opioid, n (%)^†^	
0-5%	5 (20)
6-15%	13 (52)
16-25%	6 (24)
>25%	1 (4)

* Physicians provide longitudinal care in the 3 study practices, while nurse practitioners see 'walk ins' and cover for physicians, but do not provide longitudinal care.

^†^N = 25 for these items.

### Providers' Opioid Prescribing Practices and Attitudes

Table 2 presents information on participants' practices and attitudes about prescribing opioids to older patients with chronic pain. Twenty-five of the 26 participants prescribed opioids as a treatment for chronic pain in at least some of their older patients. Of these, all but one indicated doing so cautiously. There was unanimous agreement that opioids were not first-line treatments. Most providers used phrases such as 'cautious' or 'hesitant' when describing their opioid prescribing practices in older patients. Despite these concerns, a significant minority (42%) related that opioids were effective when used in the "right" older patient. As one participant reported:

**Table 2:** Attitudes and practices regarding opioid prescribing in older adults

	Participants mentioning (N = 26) n (%)
**Attitudes and practices**	
Does not consider opioid to be first-line treatment	26 (100)
Prescribes to older adults as treatment for chronic pain	25 (96)
Uses cautiously or hesitantly	24 (92)
Endorses greater comfort prescribing to palliative care patients	19 (73)
Viewed as effective therapy for certain older patients	11 (42)
**Factors that increase likelihood of prescribing**	
Reliable patient and reliable caregiver in the home	12 (46)
Identifiable etiology of pain	8 (31)
History of beneficial results with opioid use in the past	5 (19)
**Factors that decrease likelihood of prescribing**	
Patient is unreliable or no caregiver in the home	13 (50)
Cognitive impairment	8 (31)
Polypharmacy or impaired drug metabolism	4 (15)

These medications work when given to the right patient, those who can understand the regimen and can anticipate the side effects. I have many people, one of whom was here today, who are getting relief from it and I think being able to live a life because of it.

Participants reported considering numerous patient factors when deciding whether to prescribe an opioid. Factors that increased the likelihood of prescribing included a reliable patient, the presence of a reliable caregiver in the home, an identifiable etiology of pain, and a history of benefiting from opioids in the past (Table 2). Conversely, providers stated they were far less likely to initiate a trial of an opioid therapy if the provider did not feel the patient was reliable, if the patient did not have a reliable family member or caregiver in the home, and when cognitive impairment, polypharmacy or impaired drug metabolism was present.

### Provider Barriers to Opioid Use

Table 3 shows the various prescribing barriers and facilitators that participants endorsed and indicates which ones have been previously reported.

**Table 3:** Specific Barriers and Facilitators to Opioid Prescribing in Older Adults

Themes	Participants mentioning (N = 26) n (%)	Newly identified theme^*^
**Provider-level barriers**		
Fear of causing harm from adverse effects	20 (77)	No^4,6,7,12^
Subjectivity of pain	16 (62)	No^11,24^
Lack of education in pain management	9 (35)	No^5,9,12-14^
Problem converting or dosing opioids	8 (31)	No^5^
Concern for abuse, addiction or dependence	5 (19)	No^4-12^
Concern for legal/regulatory sanction	3 (12)	No^5-7,9,10^
Concern for family member/caregiver abuse	3 (12)	Yes
**Patient/family-level barriers**		
Older patients reluctant to take opioid	18 (69)	Yes
Stigma	15 (58)	No^6^
Family reluctant to have older patient take opioid	10 (38)	Yes
Financial (medication costs)	6 (23)	No^6^
**Facilitators**		
Patient and family education about opioids	14 (54)	Yes
Studies demonstrating long-term benefit and validated risk assessment tools	11 (42)	No^12^
Easy access to peer or specialist support	7 (27)	Yes
Evidence-based tools to help calculate starting dose	3 (12)	Yes

*Studies focused on barriers in adult populations with chronic pain (not older adult populations).

#### Fear of causing harm

The most commonly reported barrier to initiating opioid therapy (reported by 77% of participants) was a fear of causing harm or that the potential harms outweighed the potential benefits (Table 3). As one physician described:

I just have a hard time prescribing opioids in my older patients. I get frightened with 80+ year olds; how are they going to respond? Am I going to absolutely drop them to the floor even with a small dose?

Fear of causing harm was often related to previous clinical experiences:

One of my 96 year-old female patients got an opioid and went to sleep for three days after taking it. It really clouded her sensorium, so that was a negative experience for her as well as for me.

Related to the fear of causing harm was the guilt some physicians experienced (or might experience) on account of opioid-related adverse events, causing them to think carefully before prescribing an opioid:

If something does happen to them, you feel guilty and want to crawl under a table when they're in the emergency room and you get the call that they fell while on the fentanyl patch you gave them. That kind of experience is powerful and definitely factors into the equation.

#### Pain subjectivity

Another frequently cited barrier (by 62%) was the subjectivity of patients' pain reports, which was often associated with an inability to establish an organic cause of pain. Providers remarked that the subjective complaint of pain was sometimes a manifestation of some psychosocial problem:

I have a lot of patients who come in and always have pain, and there is no physical reason for it; I think much of it is psychological.

Other providers remarked that patients' pain complaints/pain scores often did not correspond with their behavior during the visit, which contributed to provider skepticism about the amount of pain patients were experiencing and reluctance to prescribe opioids:

[I have patients who report that] their back hurts all the time, but then they jump right up onto the exam table, I don't get that.

Or as another physician recounted:

If I were in 10 out of 10 pain, I would be crying. I would be either in a fetal position, unable to make noise or crying hysterically and screaming. To me, 10 out of 10 is the worst pain I could imagine. And so it's really hard not to judge your patient who tells you, "Oh my pain's 10 out of 10 for sure, but the first thing I want to talk to you about is the fungus on my toenails because they're ugly."

#### Concerns about regulatory and/or legal sanctions

Of the 23 physician participants, only 9% reported concerns about possible legal or regulatory sanctions as a consequence of prescribing opioids as treatment for chronic pain. However, all three nurse practitioners voiced substantial apprehension about possible sanctions because of writing a high volume of prescriptions, often refilling prescriptions on days the patient's physician was not present in the practice.

#### Concerns about opioid abuse, misuse or addiction

Only one participant voiced concerns about the possibility of older patients abusing opioids, while four shared concerns about older patients using the medication to treat non-pain symptoms or conditions (e.g., using the medication as a sleep aid), a type of medication use that all four providers characterized as opioid misuse. Three participants relayed concerns about abuse/misuse, not by patients but by their caregivers (e.g. home attendants or family members).

Eight providers reported either low (or no) concern for addiction or dependence in older adults. As one stated:

I have a patient who continues to complain about her pain. She doesn't even want to take Tylenol #3. She is in her 80s and says, "No narcotics! No, no, no! Don't give me anything like that!" I would love to give her something, but [to the patient] an addiction is an addiction. I mean she's 80-something, how addicted is she going to get?

One physician with low concern for addiction also emphasized that pain control is imperative to improving quality of life in later years and that he counseled patients saying:

Chronic pain restricts social activity and your activity choice. If pain is not as severe, you are better able to do things you want to do.

### Facilitators to Opioid Use

Table 3 also shows the factors that providers stated would make them more likely to prescribe an opioid to an older patient with chronic pain: Patient and caregiver educational interventions; well-conducted studies that demonstrate benefit, validated tools to identify high risk older patients and evidence-based methods to help calculate appropriate starting doses for older patients with comorbidities; as well as peer support, i.e., the ability to quickly consult a colleague possessing pain management expertise.

### Perceived Patient-Level Barriers to Opioid Use

A majority of providers (69%) reported that many older patients were very reluctant to take opioids because of multiple concerns, including bothersome side effects, concerns about costs, and worries about addiction. Several providers felt that patients' reluctance to try an opioid medication acted as a barrier to prescribing this class of medication to future patients:

It's [the side effects] in addition to their knowledge that these meds are addictive. That might make it so that even when we get over our barriers to giving them, there are barriers on the patient side, and that reinforces [the notion that] they don't want it anyway. So, I'm not going to bother offering it to the next patient [who presents with significant pain].

Twelve percent of participants also reported that older patients' family members functioned as a barrier at times by voicing their own concerns about having the patient take an opioid. More than half (58%) reported that "stigma" was a barrier, causing many patients to avoid opioid therapy altogether. As one physician working in a community health center noted:

Patients hear the word codeine or some [other opioid] that they recognize and they think of it as a street drug, and don't want to be associated with that. I think in this population, when street crime is so rampant, and they have families who have been hurt by street crime or family members who are in jail because of selling, patients are very hesitant.

Stigma could also lead patients to stop taking an opioid medication altogether. Another physician relayed that:

Patients have told me, they're respectable people mind you, that they would go into the pharmacy and [the pharmacist] would yell out "we don't have any methadone" in front of all the people waiting in the pharmacy.

### Differences in Identified Themes Across the Practices

An analysis of the themes by practice site revealed that stigma was the only theme to be more frequently endorsed by providers in practices serving Hispanic patients. All remaining themes were equally endorsed across the 5 practice sites.

### Other Themes

#### Greater comfort using opioids in patients receiving palliative care

Seventy three percent of the sample stated that they were much more comfortable prescribing opioids to patients receiving palliative or hospice care, as compared to patients receiving treatment for chronic pain. Reasons included the indefinite period of treatment (for patients with chronic pain), divergent goals of care (i.e., comfort versus pain control with maintenance of function) and a different perceived risk-benefit ratio for the two groups. As one physician confided:

It might be that you are treating for a limited amount of time, just like acute pain, you're just getting them through the last few weeks, months, or few years... so maybe we feel more comfortable [treating palliative care patients with opioids], whereas the patient with chronic back pain you don't how long you're going to be treating him or her, the patient can be 60 and treated for 35 years with narcotics, so yes, I think it's a risk-benefit issue.

#### Frustration treating pain in primary care

While not related directly to opioid use in older adults, a theme of frustration treating patients with pain was identified. As one of the 8 physicians who endorsed this theme described:

Treating pain is one of the most frustrating things we do, there's a lot of pressure to treat pain, I mean we do the pain scale, we're mandated to do the pain scale, every time I go to a continuing medical education class, they're always telling me how little we treat pain, but the problem is, we don't know how to treat pain. And so everybody is telling me I'm not treating pain well, but nobody is helping me figure out how to treat the pain.

## Discussion

This investigation focused on primary care providers attitudes' and practices regarding opioid use as a treatment for chronic pain in older adults. Our study extends research in this area by documenting new prescribing barriers and facilitators, and highlights interventions that could, if implemented, possibly improve appropriate use of opioid therapy. First, older patient and family reluctance to try an opioid medication was perceived to be a major barrier. Many physicians described cases where they were unable to overcome patients' reluctance to try an opioid medication, whereas others recounted having patients in substantial pain who took the prescription, but would not fill it. Furthermore, almost 40% of providers reported that they had encountered resistance from a family member/caregiver when attempting to prescribe an opioid medication. Over half of all participants felt that interventions that educated patients and family members about the benefits and risks of opioid analgesic (relative to other classes of analgesic medications) could facilitate use of opioids in this age group. Establishing the long-term risks and benefits of therapy and generating validated tools to estimate risk for adverse outcomes were also judged to be important facilitators of future opioid use.

This study is also the first to document that opioid abuse/misuse by family members or caregivers constitutes a potential prescribing barrier. This concern was endorsed by 12% of providers. According to a recent study [21] friends, family and elderly are a significant source of opioid diversion. Older people may be at greater risk for diversion because of declining cognition, dependence on caregivers and economic hardship, making this an important issue for providers to be aware of when prescribing opioids to older patients. These findings, coupled with prior research showing that older adults are known to store their medications in insecure locations [22] and that opioid use is increasing nationally [23] supports future efforts to determine whether and to what extent older adults constitute an important route for opioid diversion.

Our study suggests that the primary barrier to prescribing opioid analgesics among older adults with chronic pain is a fear of causing harm. A recently published guideline for managing chronic pain in older adults [1] encourages clinicians to consider using opioid therapy, but acknowledges that this recommendation is based on medium- to low-quality evidence. Indeed, a recent observational study that examined analgesic-related risks in an exclusively older population of patients [3] found that participants who were prescribed opioids had a significantly increased risk of fracture, hospitalizations, and all-cause mortality compared to those prescribed non-selective non-steroidal-anti-inflammatory drugs (NSAIDs) or selective NSAIDs. This study had limitations, including an inability to account for actual consumption of prescribed medications and concurrent use of over-the-counter analgesics. In addition, clinical experience suggests that opioid therapy is typically the last pharmacotherapy to be tried, so it stands to reason that patients who received opioids were sicker at baseline with more functional impairment. Overcoming the 'fear of causing harm' barrier will likely require studies that confirm the long-term efficacy of this class of medications and practical strategies for minimizing attendant risk.

Prior investigations [11,24] suggest that pain subjectivity constitutes a strong barrier to opioid prescribing; this finding was confirmed in the current study. Many providers doubted patients reports of pain, as illustrated in statements such as "there is no physical reason for it; I think much of it is psychological." This suggests incomplete provider understanding of the etiology of chronic pain and inadequate training in what is known about its clinical presentation. One study [11] demonstrated that physicians who believe that chronic pain always has a physical cause were more likely to report prescribing opioids for chronic pain. However, with chronic pain there is often no identifiable organic cause [25,26], which contributes to uncertainty around whether and how best to intervene. These findings support future educational interventions that seek to improve provider training in assessing and managing chronic pain.

Our results also differ from prior related studies in several important ways. First, prior investigations [4-14], which focused on attitudes and barriers to opioid prescribing in adult (but not exclusively older adult) populations, found that provider concerns about opioid abuse and addiction constituted a major prescribing barrier. In the current study, few providers voiced this concern. One possible explanation for this difference is an age cohort effect. Indeed, prior research [27-30] has documented that advancing age is associated with significantly decreased risk for abuse/misuse of opioid medications. In addition, concerns about legal/regulatory sanctions constitute a frequently reported barrier to opioid prescribing [5-7,9,10], but were infrequently endorsed in the current study. This discrepancy may be explained by a lower prevalence of opioid analgesic prescribing in the study practices (relative to other study sites) or that the likelihood of administrative sanction by a state board is perceived to be lower in New York relative to other states.

Managing older patients with chronic pain is frequently a complex and challenging task, which is often compounded by feelings of helplessness and an inability to optimally manage pain on the part of healthcare providers [31,32]. Many providers in this study expressed significant frustration treating patients with chronic pain. This theme was endorsed by participants with relatively few years of clinical experience, as well as from those with many years of experience. Providers attributed their frustration to the previously mentioned barriers, particularly the subjectivity of pain, along with the pressure of mandates to assess and treat pain. Mandates and guidelines put in place to improve pain management among older adults will likely continue to contribute to frustration if the barriers that providers experience are not successfully addressed.

What types of research/initiatives could help to address the frustration that a significant minority of providers endorsed regarding managing patients with chronic pain? Providers viewed future studies that ascertain the long-term safety and efficacy of these drugs and how different subgroups of older patients respond to varying opioid medication regimens to be particularly relevant. These data could be used to develop evidence-based guidelines and algorithms for opioid prescribing in later life. Such data, along with improved training [32], may instill in providers a greater sense of confidence when prescribing opioids to older adults. This could also help providers when counseling patients and caregivers about opioid medications, which could serve to address the barrier of patient or caregiver reluctance.

Our results also provide support for future efforts to improve provider training and access to peer support. Preliminary testing of a clinical decision support system to facilitate opioid prescribing for chronic pain in primary care is encouraging [33]. Additionally, a recent study [34] evaluated a tool to identify older patients at risk for adverse drug events, which may support development of a tool to assess risk associated with opioid use. Tools such as these may help to overcome some of the barriers identified in this and prior investigations [7,12,14], including lack of training, problems with dosage calculations, and peer support, and help to support appropriate documentation in the medical record and decrease provider frustration. Finally, it is important to acknowledge that providers, patients, and caregivers can have very different tolerance levels for risk regarding the use of medications for the treatment of pain. Generating efficient ways to solicit patients' and/or caregivers' risk levels and to have providers' acknowledge their respective concerns about level of risk these medications entail -prior to arriving at a joint decision about initiating a course of opioid therapy -should also be a focus of future work.

There are several limitations to this study. Although all participants engaged in the focus group discussions, because of group dynamics and the participants' relationship as colleagues, these data may not have accurately captured the full extent of participants' views on this topic. Additionally, the actual prescribing patterns and clinical impact of the perceived barriers are unknown. We did not formally calculate an index of inter-coder agreement when analyzing our qualitative results. However, disagreement between raters occurred rarely when coding the themes reported in this paper, indicating that overall inter-rater agreement was high. The sample was small, non-random, and limited to providers at two academically affiliated and three community-based primary care practices in New York City. Finally, most providers cared for predominantly older adults of White or Hispanic origin. Our findings may not generalize to other race/ethnicity groups or non-elderly populations.

## Conclusions

In conclusion, this study demonstrated that primary care providers perceive multiple barriers to the use of opioids for older adults with chronic pain and use opioids cautiously for this purpose. Newly identified barriers include older patient/family member reluctance to take an opioid and the possibility of opioid abuse by caregiver (e.g., family members and home attendants). Further work directed at addressing the identified barriers and optimal ways to facilitate decision making when tolerance levels for risk vary between providers and patients/caregivers will be needed.

## Authors' contributions

AS assisted with study design, collected data, conducted analysis and interpretation, and drafted the manuscript. AAM conceptualized and designed the study, and commented on the manuscript. MP conceptualized and designed the study, collected data, and commented on the manuscript. EG conceptualized and designed the study and commented on the manuscript. BJT conceptualized and designed the study, and commented on the manuscript. MCR conceptualized and designed the study, collected, analyzed and interpreted data, and drafted the manuscript. All authors read and approved the final manuscript.

## Competing interests

The authors declare that they have no competing interests.

## Pre-publication history

The pre-publication history for this paper can be accessed here:

http://www.biomedcentral.com/1471-2318/11/35/prepub
